# Supplementary figures and images for: Multisensory gamma stimulation enhances adult neurogenesis and improves cognitive function in male mice with Down Syndrome
Source: PLoS One. 2025 Apr 24;20(4):e0317428. doi: 10.1371/journal.pone.0317428 (PMC12021272; doi:10.1371/journal.pone.0317428)

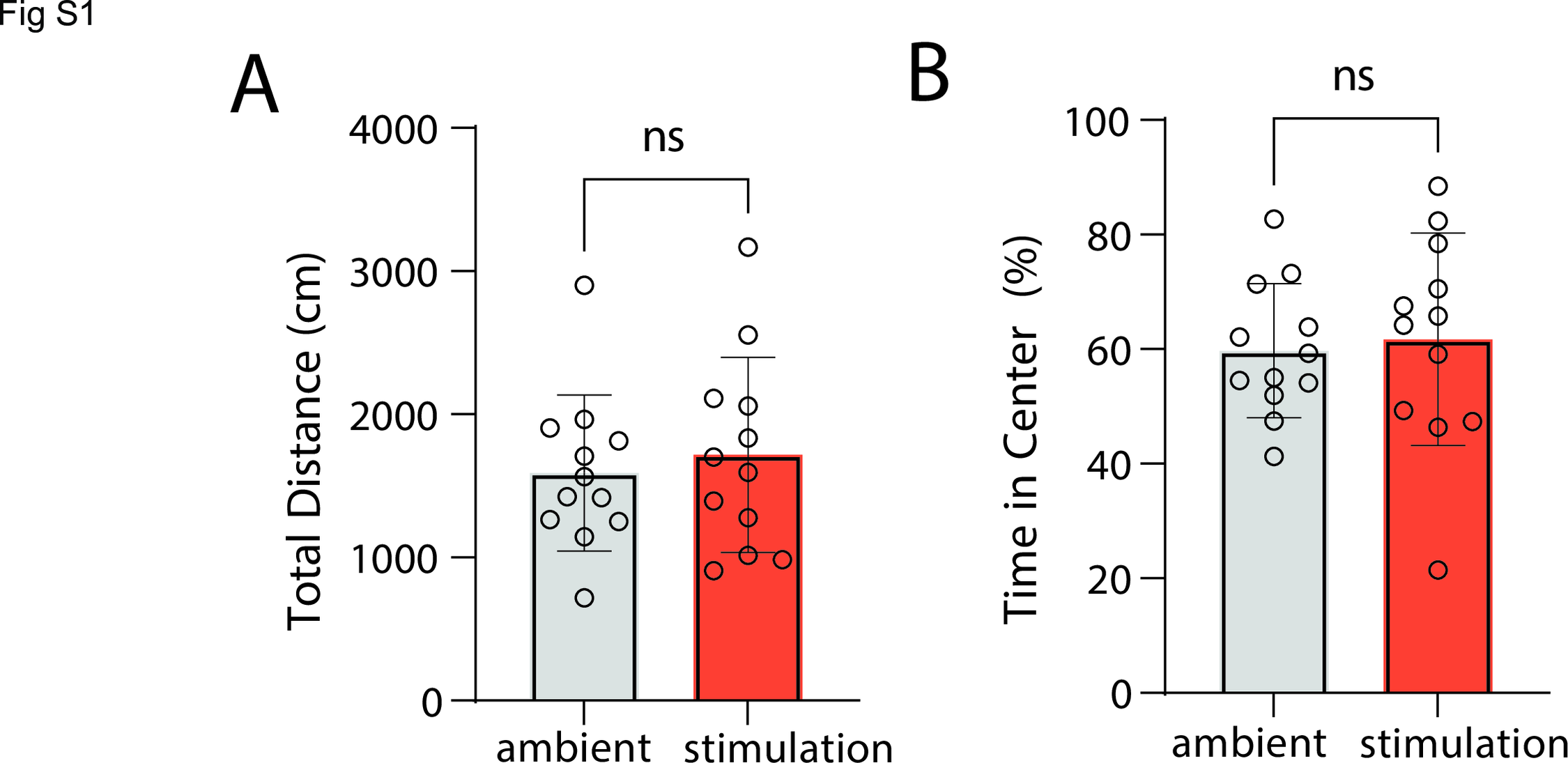

Supplement: S1 Fig — A) Quantification of total distance traveled during open field test B) Quantification of time spent in the center. Error bars indicate mean ± standard deviation, unpaired t-test, two-tailed, ambient = 12, stimulation = 12. (TIF) [file pone.0317428.s001.tif]

Fig S2

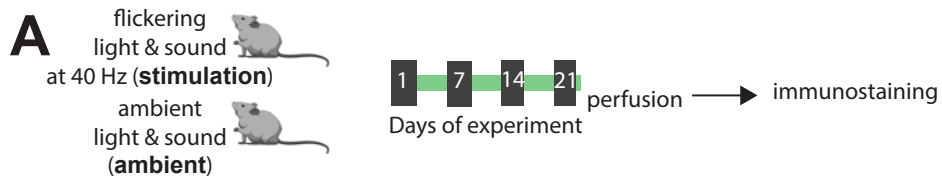

**B**

**ambient**

**stimulation**

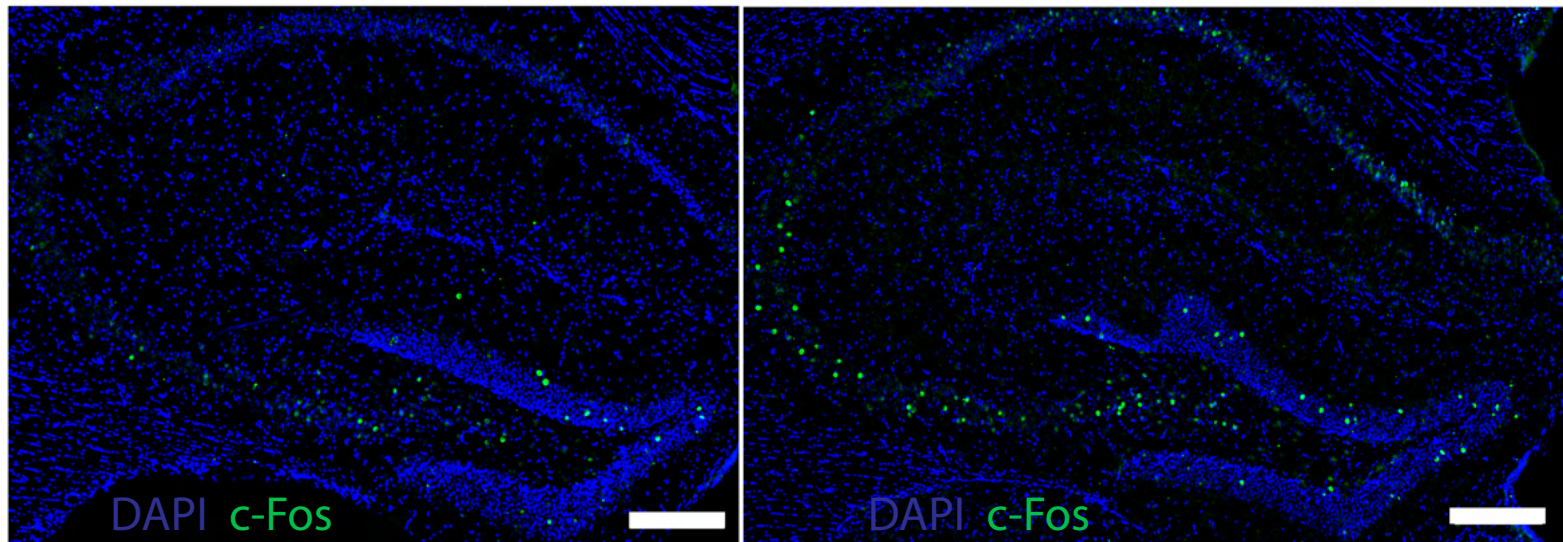

**C**

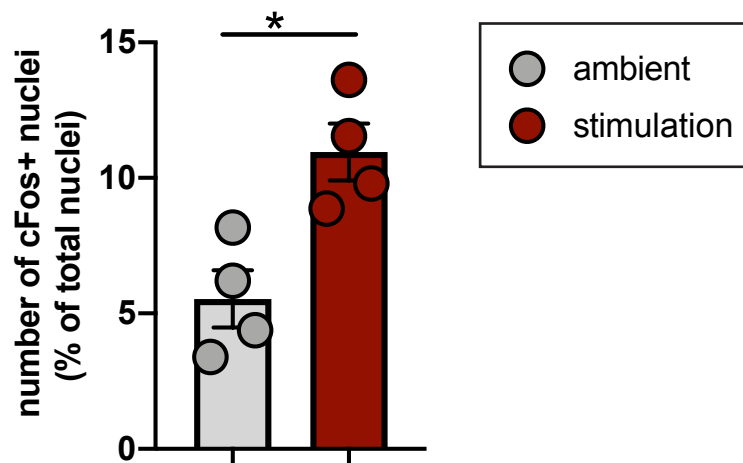

Supplement: S2 Fig — A) Experimental outline. B) Representative images of c-Fos+ nuclei in the hippocampus after ambient or 40 Hz stimulation. Blue represents DAPI and green represents c-Fos signals. Scale bar = 200 µm. C) Bar plots showing the percentage of c-Fos+ nuclei in the two groups. N = 4 mice/group. Error bar indicates mean ± sem. Two-tailed, unpaired t-test, *P<0.05. (PDF) [file pone.0317428.s002.pdf]

Fig S3

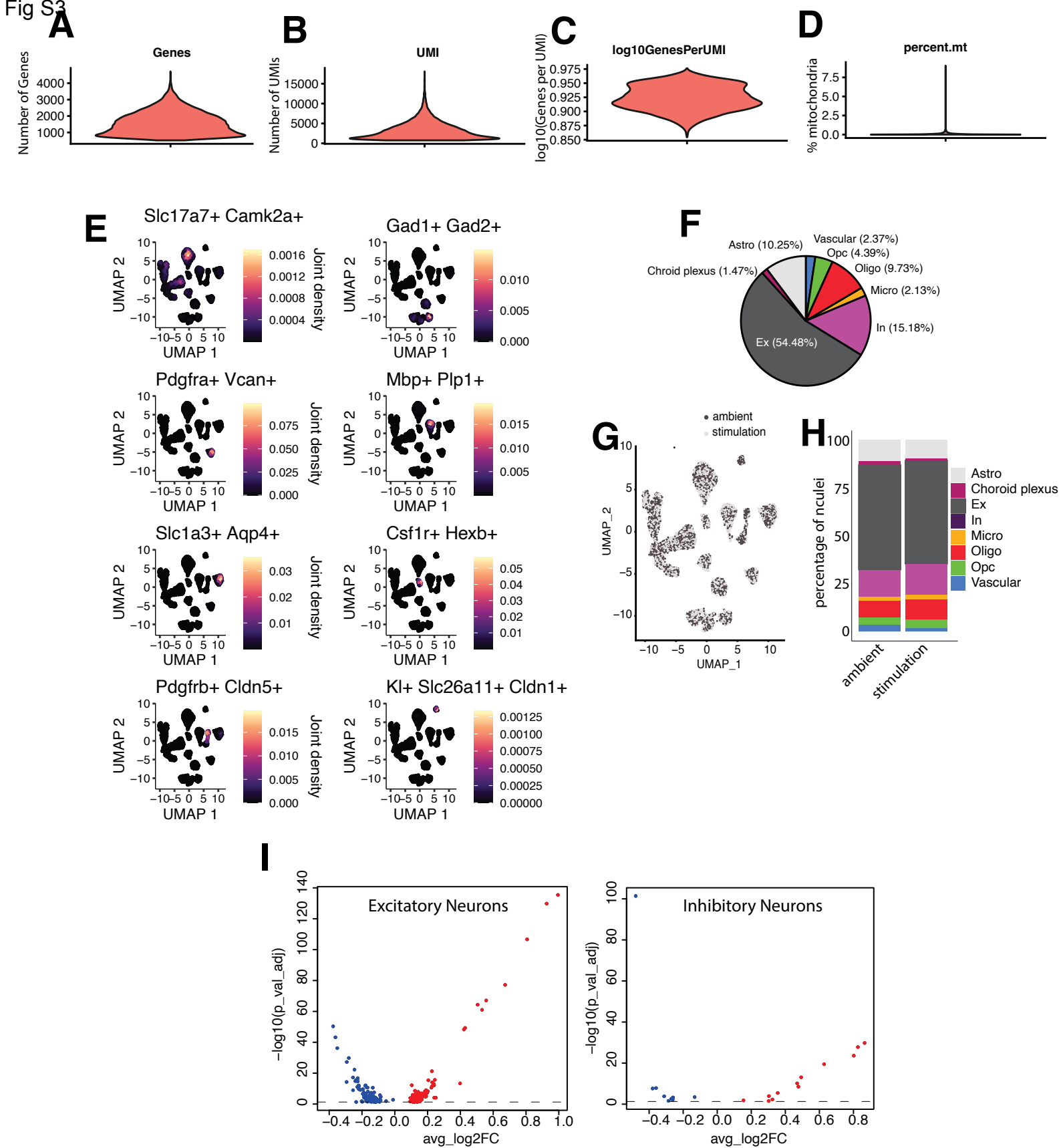

Supplement: S3 Fig — Distribution of A) number of genes B) number of UMI, C) complexity and D) mitochondrial percentage across 15884 nuclei (ambient: 7665, stimulation: 8219). E) Canonical cell type specific marker expression in cognate cell types. Slc17a7, Camk2a in excitatory neurons; Gad1, Gad2 in inhibitory neurons; Pdgfra, Vcan in OPC; Mbp, Plp1 in Oligodendrocytes; Slc1a3, Aqp4 in Astrocytes; Csf1r, Hexb in microglia; Pdgfrb, Cldn5 in vascular cells, KI, Slc26a11, Cldn1 in choroid plexus cells. F) Pie chart displaying the distribution of detected cell types. G) Nuclei from stimulation and ambient Ts65Dn mice groups are well integrated. H) Proportion of nuclei detected across cell types in both groups. I) Volcano plots showing genes that are significantly altered in excitatory and inhibitory neurons in the stimulation group compared to the ambient group. (PDF) [file pone.0317428.s003.pdf]

Fig S4

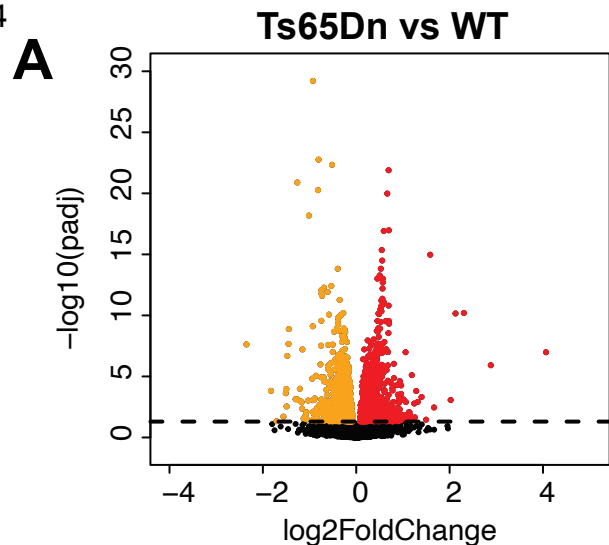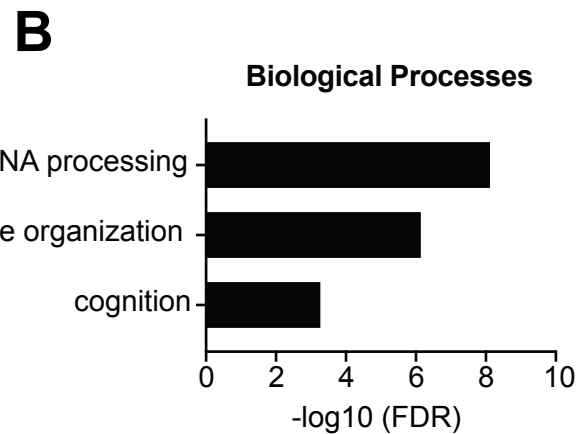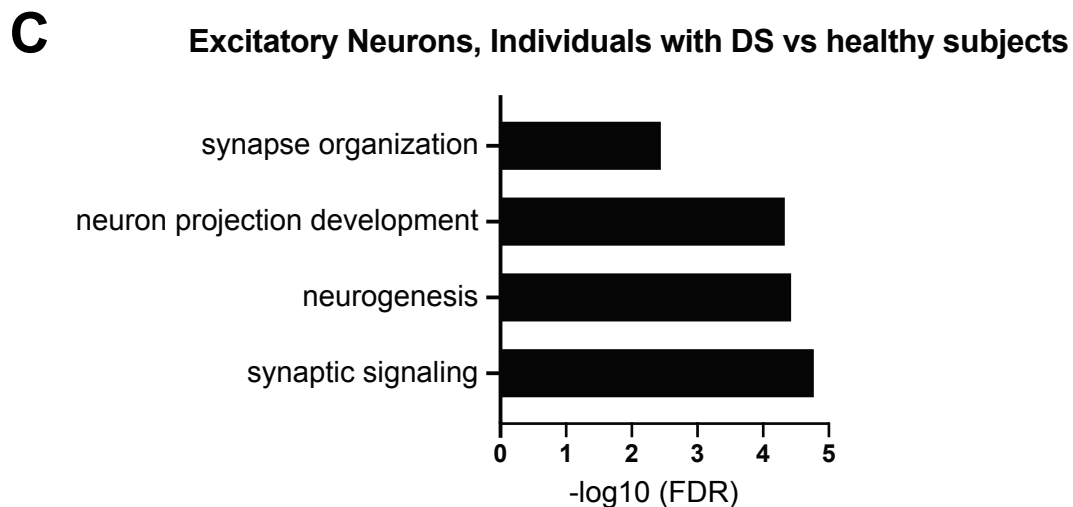

Supplement: S4 Fig — A) Differential gene expression observed in bulk RNA-seq data from hippocampi and cortices of Ts65Dn mice (n = 6) and littermate controls (n = 6). Data were retrieved from GSE213500. Gene counts were normalized and differential expression analysis was performed using DESeq2 after adjusting for region specific difference. B) Gene ontology analyses revealed that the genes related to mRNA processing, synapse organization and cognition are downregulated in Ts65Dn mice compared to the controls. C) GO analysis of genes downregulated in excitatory neurons of post mortem brains of individuals with DS compared to healthy subjects. Gene expression changes data were retrieved from Palmer CR et al61. (PDF) [file pone.0317428.s004.pdf]

Fig S5

**A**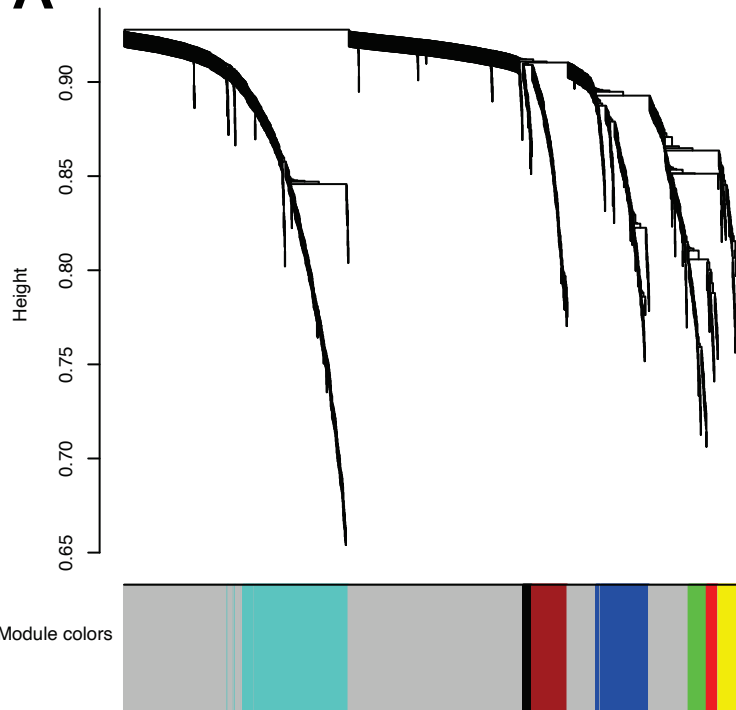**B**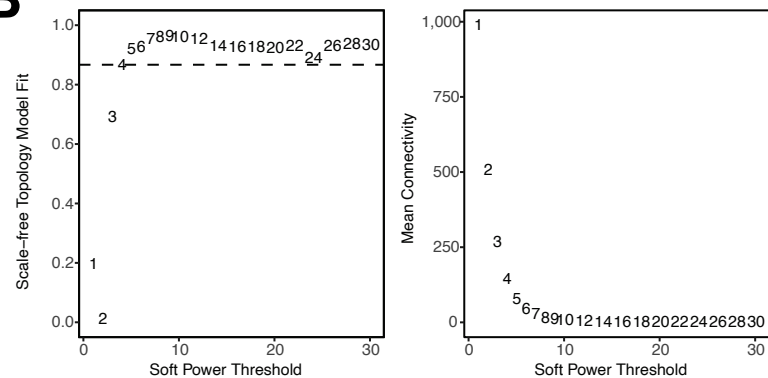**C**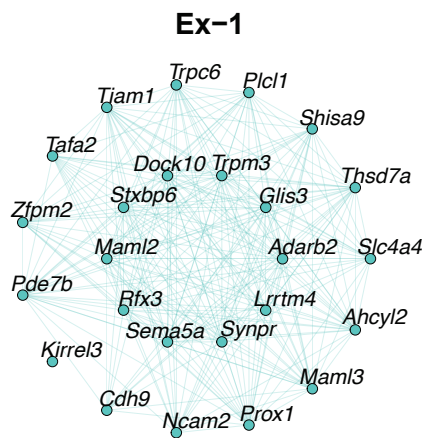**Ex-2**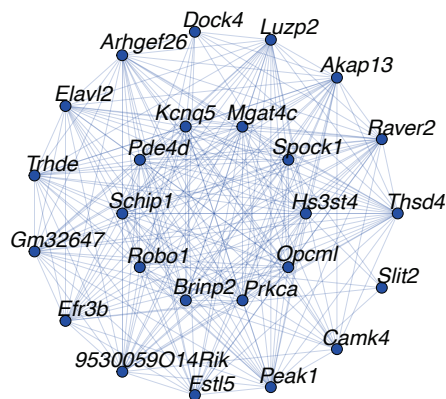

Supplement: S5 Fig — A) Cluster dendrogram for seven excitatory gene modules. B) soft-power threshold was determined based on scale-free topology measure and mean connectivity (see Methods). C) Hub genes of Ex-1 and Ex-2 module. (PDF) [file pone.0317428.s005.pdf]

Fig S6

ambient

stimulation

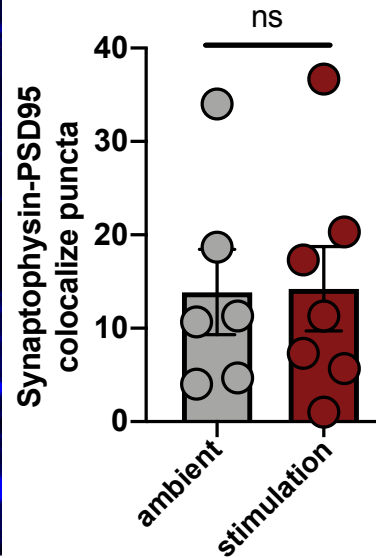

Synaptophysin  
PSD95  
DAPI

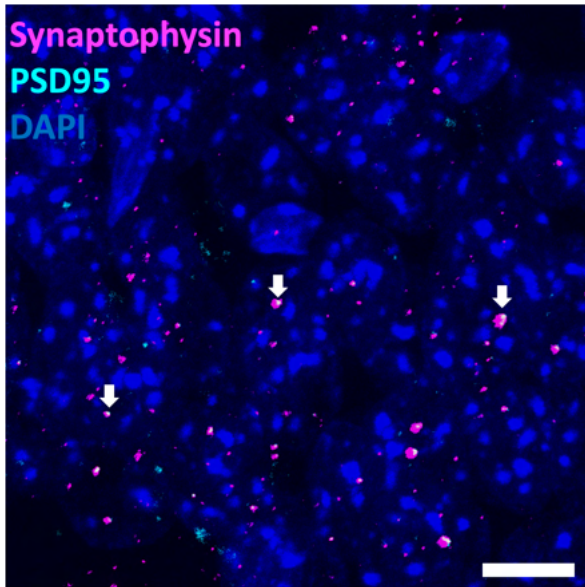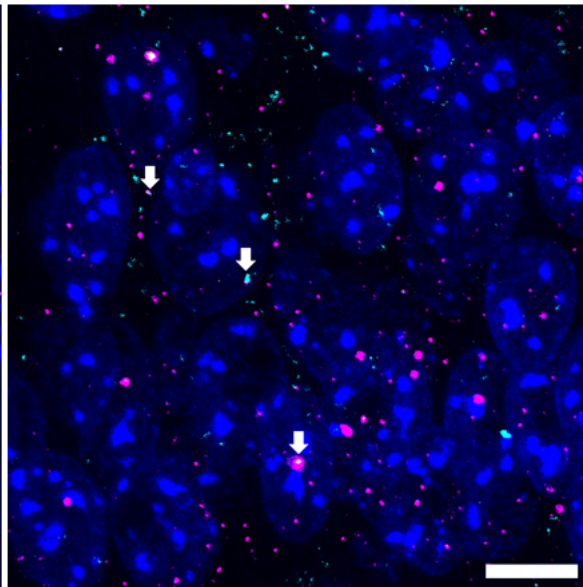

Supplement: S6 Fig — (Left) Representative images showing PSD95 and Synaptophysin co-localization. White arrows indicate colocalized puncta. Scale bar = 10 µm. (Right) Bar plots showing the absolute number of colocalized puncta. Statistical significance was determined using a two-tailed, unpaired t-test, N = 6–7 per group. Error bar: mean ± standard error mean. (PDF) [file pone.0317428.s006.pdf]

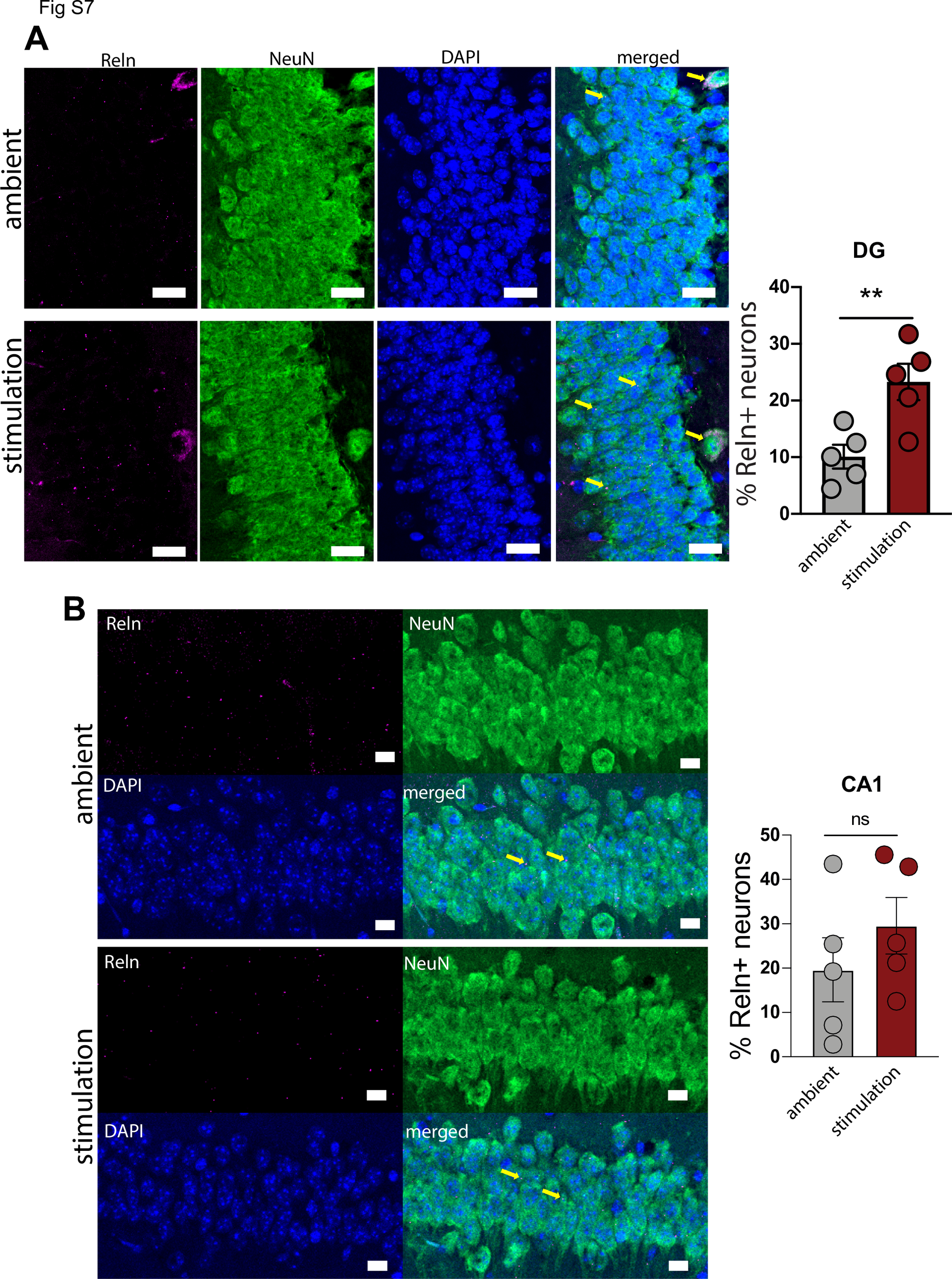

Supplement: S7 Fig — A) (Left) Representative images of Reln+ neurons in the DG granule cell layer of the hippocampus. (Right) Barplots show percentages of Reln+ neuorns between two groups. 40 Hz stimulation increased percentages of Reln+ neurons in DG of DS mice. Scale bar = 20 µm. Two-tailed t-test, **P<0.01, N = 5 per group. B) (Left) Representative images of Reln+ neurons in the hippocampal CA1 region. (Right) Barplots show percentages of Reln+ neuorns between two groups. Scale bar = 10 µm. Two-tailed, unpaired t-test, N = 5 per group. (TIF) [file pone.0317428.s007.tif]

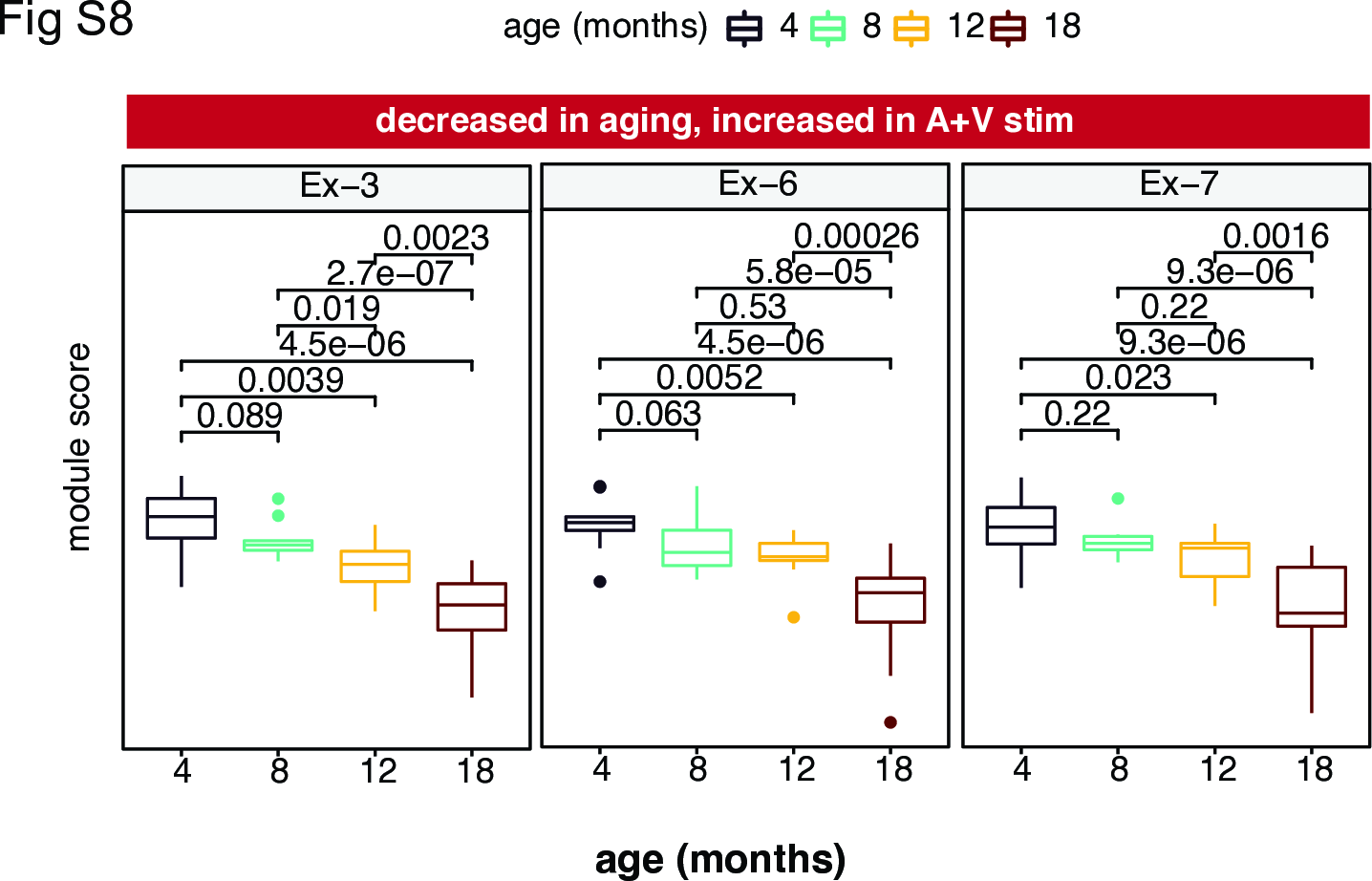

Supplement: S8 Fig — Expression patterns of module Ex-3, Ex-6 and Ex-7 display reduced co-expression at old age (18 months). Boxplots show changes in gene module expression along aging in the hippocampus. Y-axis shows gene module expression eigenvalue. In the box plots, median is marked with the center line, while the lower and the upper lines represent the 25th and 75th percentiles, respectively. The whiskers indicate the smallest and largest values respectively in the 1.5x interquartile range. P-value determined by Kruskal-Wallis test. (TIF) [file pone.0317428.s008.tif]

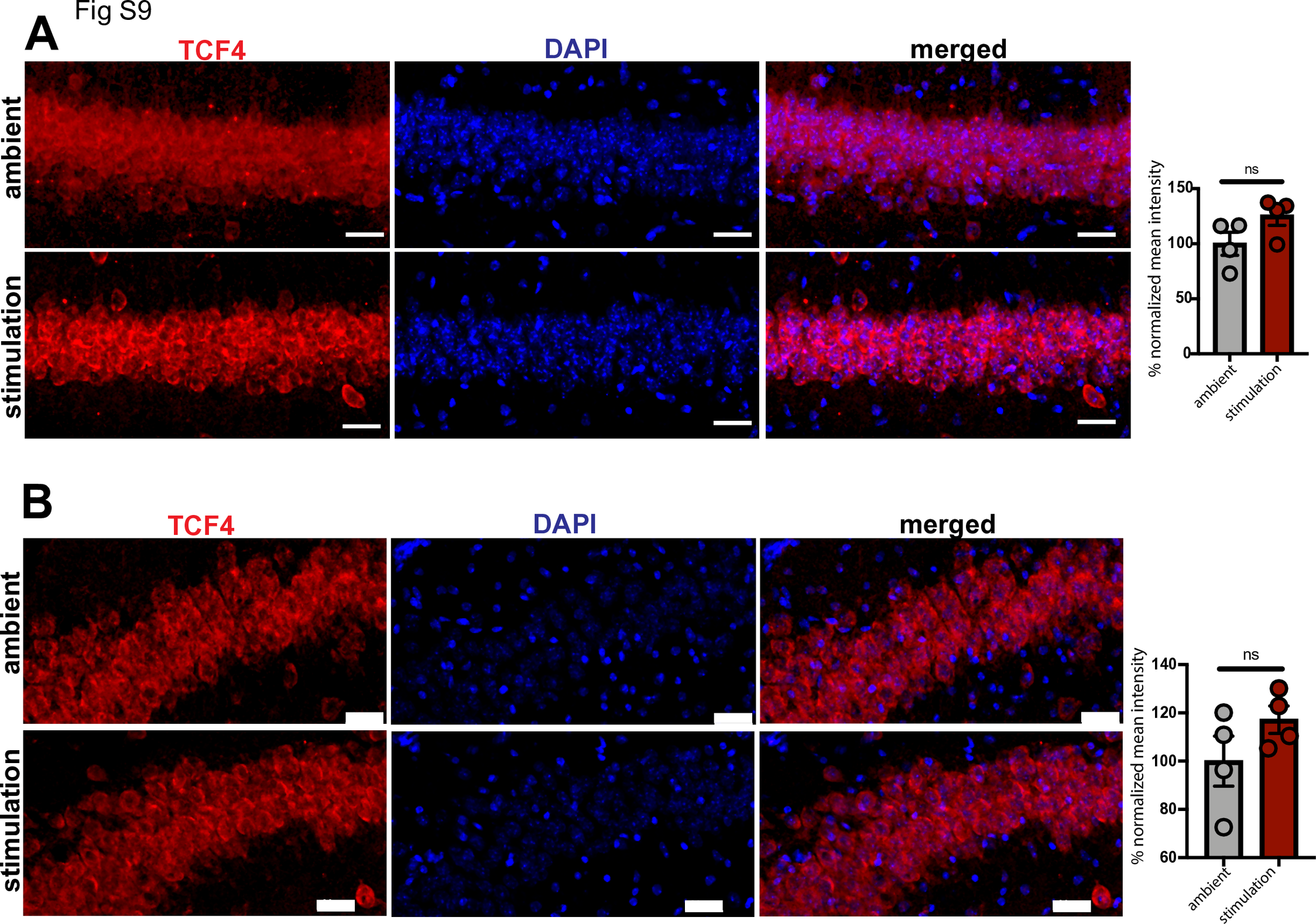

Supplement: S9 Fig — A) (Left) Representative images of TCF4 expression in the CA1 region of the hippocampus. (Right) Bar plots showing normalized mean intensity (% of the ambient group) between the two groups. Two-tailed, unpaired t-test, N = 4 per group. Scale bar = 30 µm. B) (Left) Representative images of TCF4 expression in the CA3 region of the hippocampus. (Right) Bar plots showing normalized mean intensity (% of the ambient group) between the two groups. Two-tailed t-test, N = 4 per group. Scale bar = 30 µm. (TIF) [file pone.0317428.s009.tif]

Fig S10

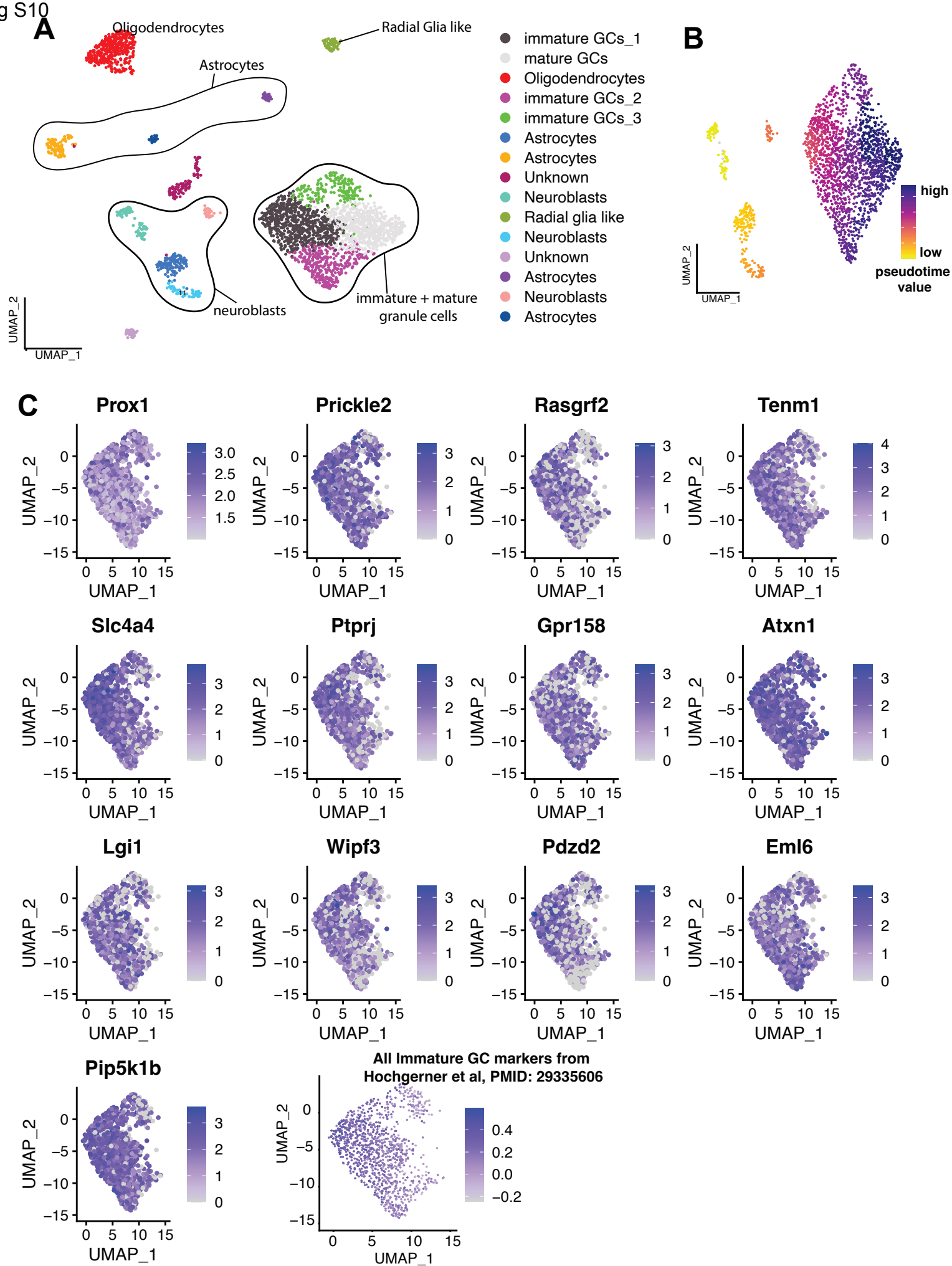

Supplement: S10 Fig — A) Prox1+ hippocampal nuclei were sorted in silico, clustered, and visualized on a UMAP. Cell clusters were annotated based on marker genes. Neuroblasts and GCs were selected for downstream analyses. B) Pseudotime analysis was performed to gain better insights into granule cell maturation. Cells are color coded based on the inferred progression along the maturation trajectory. The color code represents the relative pseudotime values, with yellow indicating cells at the earlier stage of maturation and dark blue indicating cells at the mature stage. C) Immature GC-specific markers were used to further confirm the presence of immature GCs. (PDF) [file pone.0317428.s010.pdf]

Fig S11

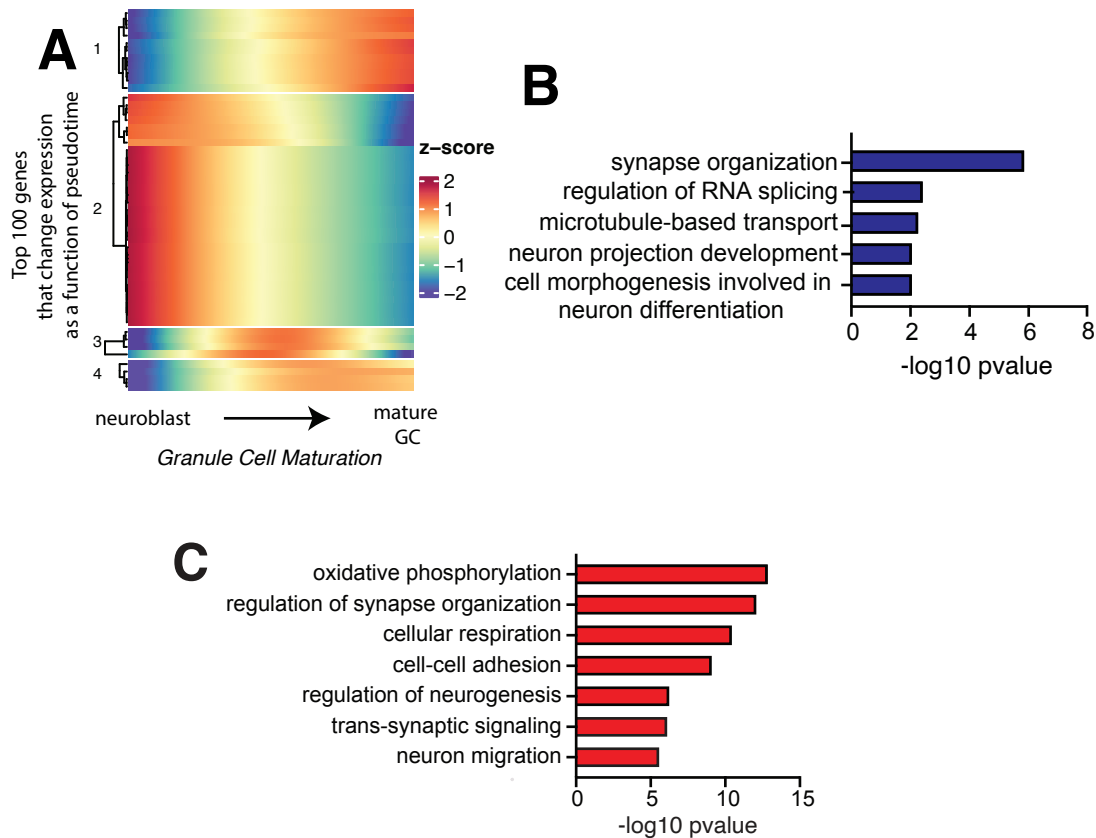

Supplement: S11 Fig — A) Heatmap showing relative expression of top 100 genes that significantly changed (q<0.05) along the maturation trajectory of granule cells (GC) in stimulation vs ambient Ts65Dn mice B) Biological processes representing differentially expressed genes (up and down-regulated) along the maturation trajectory of GC between the stimulation and ambient groups. C) Biological processes for genes differentially expressed between cluster 1,3 and cluster 2,3. (PDF) [file pone.0317428.s011.pdf]
